# Combined Approach with Ultrasound and Pressure–Volume Loops to Manage Mechanical Ventilation in Acute Hypoxemic Respiratory Failure

**DOI:** 10.3390/diagnostics16203339

**Published:** 2026-10-14

**Authors:** Javiera Aguilera Scarpati, Diana Adrião, Pablo Mercado, Michel Slama, Nello De Vita, Rosanna Vaschetto, Marco Pozzi, Belaïd Bouhemad, Silvia Mongodi, Francesco Mojoli

**Affiliations:** 1 Physical Medicine and Rehabilitation Unit, Clínica Universidad de los Andes, Santiago 7650568, Chile; javiera.scarpati@gmail.com; 2 Department of Intensive Care, Unidade Local de Saúde Gaia e Espinho, 4434-502 Vila Nova de Gaia, Portugal; diana_adriao@hotmail.com; 3 Faculty of Medicine, University of Porto, 4200-319 Porto, Portugal; 4 Departamento de Paciente Crítico, Clínica Alemana de Santiago, Facultad de Medicina, Universidad del Desarrollo, Santiago 7650568, Chile; pablovmercado@gmail.com; 5 Medical Intensive Care, University Hospital Amiens, 80054 Amiens Cedex, France; slama.michel@chu-amiens.fr; 6 Department of Translational Medicine, Eastern Piedmont University, Via Paolo Solaroli, 17, 28100 Novara, Italy; nellodevita@hotmail.com (N.D.V.); rosanna.vaschetto@med.uniupo.it (R.V.); 7 Anesthesia and Intensive Care, Ospedale Maggiore Della Carità, 28100 Novara, Italy; 8 Rianimazione 1, Fondazione IRCCS Policlinico San Matteo, 27100 Pavia, Italy; 9 Lipides Nutrition Cancer—UMR 866 INSERM 1231, Université Bourgogne Franche-Comté, 21709 Dijon, France; belaid_bouhemad@hotmail.com; 10 Department of Anesthesiology and Intensive Care, C.H.U. Dijon, 21079 Dijon Cedex, France; 11 Department of Emergency and Critical Care Medicine, St. Marianna University School of Medicine, Kawasaki 216-8511, Kanagawa, Japan; francesco.mojoli@gmail.com

**Keywords:** PEEP setting, point-of-care ultrasound, pressure–volume loop, ARDS, personalized mechanical ventilation, recruitment, recruitability, atelectrauma, heart–lung interaction

## Abstract

Acute hypoxemic respiratory failure (AHRF), particularly acute respiratory distress syndrome (ARDS), is characterized by marked heterogeneity in lung morphology and mechanics, requiring individualized mechanical ventilation strategies that balance adequate gas exchange with the prevention of ventilator-induced lung injury (VILI). Lung ultrasound (LUS) has become a cornerstone of critical care imaging, allowing differential diagnosis, quantitative assessment of lung aeration, phenotyping of ARDS morphology, and monitoring of disease evolution and complications. However, imaging alone does not provide direct guidance for optimizing ventilator settings. Some modern ventilators enable the rapid bedside acquisition of tidal pressure–volume (PV) loops, allowing tidal hysteresis to be measured and thereby providing complementary physiological information by identifying cyclic opening and closing of lung units and signs of overdistension. Emerging evidence supports the use of tidal hysteresis thresholds to identify insufficient positive end-expiratory pressure (PEEP) associated with tidal recruitment/derecruitment and to guide individualized PEEP titration during decremental PEEP trials. This review discusses the rationale and practical implementation of an integrated approach combining critical care ultrasound and PV loops throughout the management of mechanically ventilated patients with AHRF. We describe how ultrasound can support diagnosis, phenotyping, monitoring of lung aeration, and detection of ventilation-related complications, while PV loops provide a practical physiological framework for optimizing ventilator settings. This approach may provide a comprehensive, non-invasive, and repeatable bedside assessment of lung morphology, aeration, and respiratory mechanics, potentially facilitating personalized mechanical ventilation strategies aimed at reducing the risk of VILI and optimizing patient management.

## 1. Introduction

Optimizing invasive mechanical ventilation in patients with acute respiratory failure remains a daily challenge in Intensive Care Unit (ICU) practice [1]. Personalization of the respiratory strategy seems to offer survival benefits in acute respiratory distress syndrome (ARDS) [2]. Ideally, this approach relies on multimodal respiratory monitoring, including a reliable imaging technique to phenotype the patient and monitor regional aeration, together with advanced assessment of respiratory mechanics to enable fine-tuning of ventilator settings, particularly the selection of the most appropriate positive end-expiratory pressure (PEEP) [3].

The use of ultrasound in the ICU has increased significantly over the past two decades [4]. Its widespread adoption reflects its advantages as a noninvasive, immediately available, and repeatable bedside tool, making it practical for diagnosis, clinical management, and procedural guidance [5]. Ultrasound has become a fundamental tool in the comprehensive assessment of ICU patients, and many of its applications are now considered essential skills for all intensivists [6]. In particular, lung ultrasound (LUS) has become an essential tool in the management of critically ill patients with acute hypoxemic respiratory failure (AHRF), including those with ARDS, as it allows qualitative assessment [7,8], reliable quantification of regional aeration [9], and early detection of complications [10].

Despite their accuracy in assessing the potential for recruitment or the response to prone positioning [11], imaging modalities do not provide guidance for optimizing mechanical ventilation settings at the individual patient level. The quasi-static pressure–volume loops can be easily and safely obtained at the bedside and provide a comprehensive assessment of the respiratory system mechanics [12]. Systematic measurement of tidal hysteresis during a decremental PEEP trial allows identification of the threshold at which PEEP fails to prevent lung instability (tidal recruitment/derecruitment of lung units), a principal driver of ventilator-induced lung injury (VILI) [13]. Moreover, this assessment can be performed daily as a monitoring tool, enabling adjustment of ventilator settings in response to changes in the patient’s respiratory mechanics [3,14].

Throughout this review, AHRF denotes the broader clinical syndrome, whereas ARDS refers to patients fulfilling established diagnostic criteria. Critical care ultrasound has diagnostic and monitoring applications across AHRF, but evidence supporting tidal PV loop interpretation and the proposed approach to PEEP titration derives mainly from invasively ventilated patients with ARDS and should be extrapolated cautiously to non-ARDS AHRF.

This focused narrative review synthesizes the available evidence on the use of LUS and PV loops in the management of mechanically ventilated patients with AHRF. References were selected according to their relevance to the clinical and physiological topics addressed, with particular emphasis on clinical guidelines and consensus documents, original clinical and physiological studies, and key reviews. The review presents a pathophysiology-based approach integrating proposed mechanisms and emerging evidence with their potential clinical applications, illustrated through representative clinical examples, while highlighting important gaps in knowledge that warrant further investigation.

## 2. Combined Approach with Ultrasound and Pressure–Volume Loops

### 2.1. Differential Diagnosis of ARDS Using Critical Care Ultrasound

#### 2.1.1. Lung Ultrasound Features

Computed tomography (CT) has shown that ARDS affects the lungs heterogeneously, with some areas severely affected, others mildly involved, and still others spared [15]. However, routine CT imaging is often impractical in critically ill patients and may not be readily available in many low- and middle-income settings. In this context, LUS has emerged as a valuable bedside tool for the differential diagnosis of ARDS [16]. Typical LUS findings in ARDS reflect a bilateral, heterogeneously distributed loss of lung aeration, commonly referred to as alveolar–interstitial syndrome [17,18]. These abnormalities usually have a patchy distribution, similar to CT findings, with spared areas showing normal A-lines adjacent to severely affected areas characterized by multiple or confluent B-lines. Posterior lung fields frequently show consolidations with a tissue-like pattern, particularly at the lung bases. The pleural line is frequently affected and appears irregular and thickened, often with associated subpleural consolidations and reduced lung sliding [17,18]. The distribution of these abnormalities is typically non-uniform. Pleural effusions are relatively uncommon in ARDS and, when present, are usually small and asymmetric. Because ARDS is an inflammatory process, pleural fluid may appear echogenic or flocculated. In contrast, acute cardiogenic pulmonary edema typically produces more homogeneous bilateral ultrasound findings. The alveolar–interstitial syndrome usually involves both anterior and posterior lung fields, without spared areas and often with a posterior-to-anterior gradient in the severity of ultrasound abnormalities. In the acute phase, the pleural line is generally thin and regular, with preserved lung sliding. Pleural effusions are more frequent, typically bilateral, and often larger than those observed in ARDS; because they are transudates, they usually appear anechoic and homogeneous [10,17].

#### 2.1.2. Cardiac Ultrasound Features

Diagnosing ARDS requires establishing that the respiratory failure is not primarily explained by cardiogenic pulmonary edema, although elevated left ventricular (LV) filling pressures may coexist. Accordingly, objective hemodynamic assessment—such as bedside echocardiography—is recommended to rule out hydrostatic pulmonary edema [19]. LV filling pressure can be estimated using several Doppler echocardiographic parameters. Transmitral flow assessed using pulsed-wave Doppler reflects the left atrial–left ventricular pressure gradient during diastole; however, it is influenced by multiple factors, including preload, afterload, diastolic function, and patient characteristics such as age and myocardial disease [20]. The ratio of early transmitral flow velocity (E) to early diastolic mitral annular velocity (e′), known as the E/e′ ratio, is widely used as a surrogate marker of LV filling pressure. Elevated E/e′ values (>14) are generally associated with increased left atrial pressure and higher LV end-diastolic pressure; however, this parameter has well-recognized limitations in mechanically ventilated patients since most validation data come from relatively stable patients with chronic cardiac disease [21]. Current recommendations emphasize a multiparametric echocardiographic approach that integrates mitral inflow patterns, tissue Doppler velocities, left atrial size, and pulmonary pressures to improve the differentiation between cardiogenic and noncardiogenic pulmonary edema [21,22].

#### 2.1.3. Integration of Ultrasound Findings into the Definition of ARDS

The Kigali modification of the Berlin definition replaced chest radiography and CT with LUS for the diagnosis of ARDS [23] and contributed to the development of the recent global definition [24]. Although this modification enhances feasibility in low- and middle-income countries and probably improves the early detection of ARDS compared with chest radiography, LUS criteria based on bilateral B-lines and/or consolidations may be insufficiently specific, potentially leading to overestimation of ARDS incidence [25]. Restricting the criteria to more specific LUS signs could further improve the accuracy of a clinical–ultrasound definition of ARDS.

### 2.2. Quantitative LUS and ARDS Phenotyping

**Figure 1:**
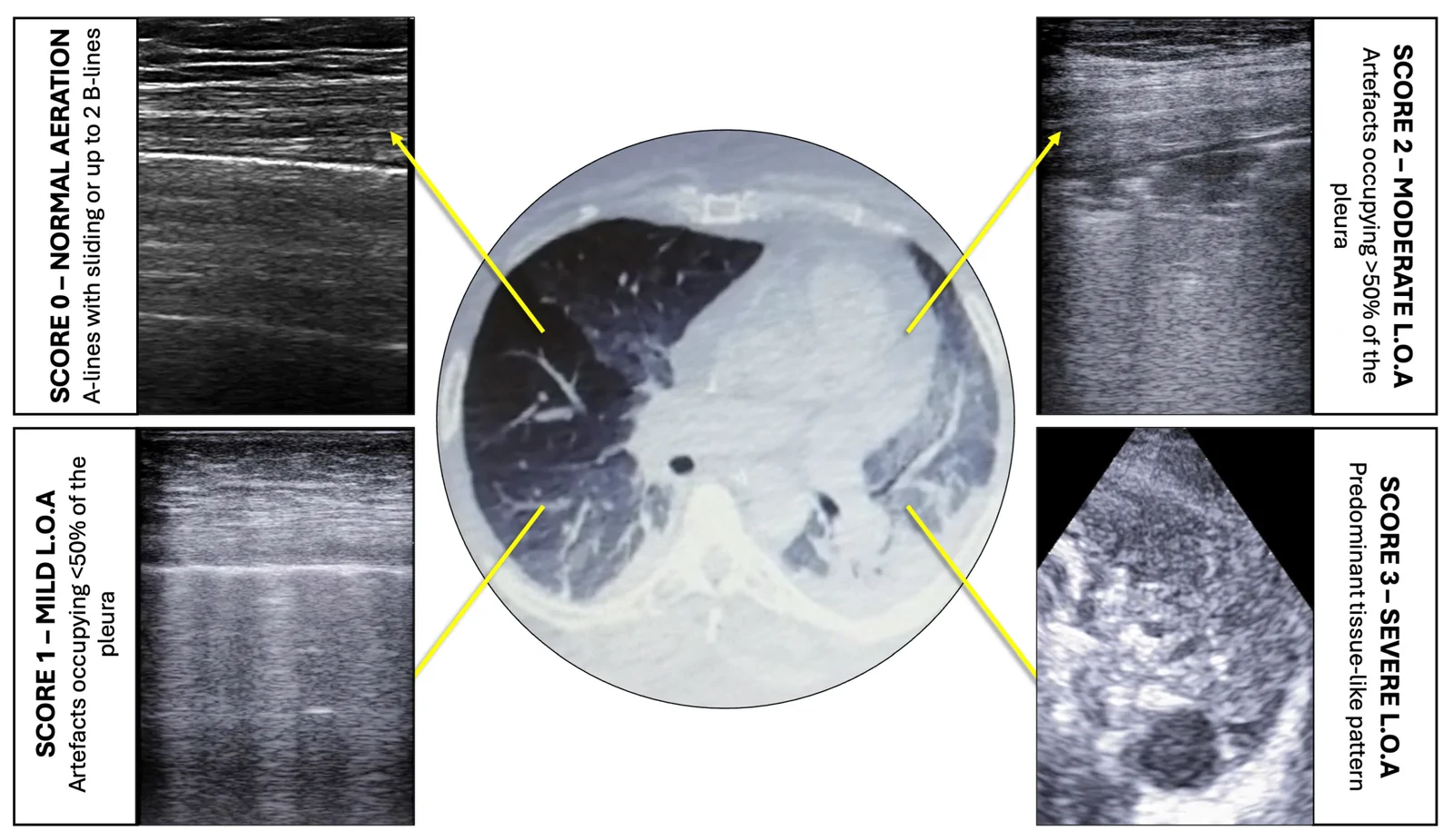
Representation of the four steps of loss of aeration and the corresponding CT findings. Score 0—normal aeration: A-lines with lung sliding are visualized; no more than two well-spaced B-lines may also be present. Score 1—mild loss of aeration: artefacts (i.e., well-spaced or coalescent B-lines or subpleural consolidations) occupy ≤ 50% of the visualized pleura. Score 2—moderate loss of aeration: artefacts occupy clearly more than 50% of the visualized pleura. In this example, a subpleural consolidation is visible in the center of the image as an echo-poor image applied to the pleural line. Score 3—severe loss of aeration: predominant tissue-like pattern (lobar or hemilobar consolidation) with a thickness > 2–2.5 cm. LOA: Loss of Aeration. Images were acquired by Vivid-IQ GE Healthcare (Chicago, IL, USA) and Esaote My Lab, Esaote SpA (Genoa, Italy).

Quantitative lung ultrasound enables estimation of both regional and global loss of lung aeration by calculating the LUS aeration score [9,26,27]. This scoring system describes four progressive stages of aeration loss (Figure 1). A score of 0 indicates normal aeration, characterized by A-lines or no more than two B-lines. A score of 1 represents mild aeration loss, defined by three or more well-spaced B-lines, coalescent B-lines, or small subpleural consolidations involving ≤50% of the pleural line. A score of 2 reflects moderate aeration loss, with multiple or coalescent B-lines and/or subpleural consolidations affecting more than 50% of the pleural line. A score of 3 corresponds to severe aeration loss and is characterized by a tissue-like pattern >2–2.5 cm in thickness, consistent with lung consolidation. The LUS score is obtained by examining 12 thoracic regions—six per hemithorax—and summing the regional scores, yielding a global score ranging from 0 (normal aeration) to 36 (complete consolidation) [11]. This method has been validated against quantitative CT imaging and extravascular lung water measurements and has shown strong correlation with lung density and aeration loss [9,26,27,28]. Higher LUS scores are associated with greater loss of lung aeration and increased disease severity [29,30].

In patients with ARDS, quantitative LUS also allows differentiation between focal and non-focal patterns [31,32]. According to the CT definition, a focal ARDS pattern is characterized by sparing of the anterior lung regions, whereas a non-focal ARDS pattern involves both anterior and posterior regions [33]. These phenotypes may require different ventilatory strategies. Patients with non-focal ARDS generally exhibit greater lung recruitability and may benefit from higher levels of PEEP. In contrast, focal patterns tend to have limited recruitability and may be more prone to overdistension when high PEEP levels are applied. A personalized ventilation strategy based on ARDS morphology (i.e., PEEP titration in non-focal phenotypes and prone positioning in focal phenotypes) has been associated with improved survival when the phenotypes are correctly identified [2]. Chest radiography has proven to be an unreliable tool for distinguishing these phenotypes and may increase the risk of inappropriate ventilatory strategies, leading to poorer outcomes. In contrast, several LUS-based approaches have demonstrated good agreement with CT in identifying ARDS morphology [32]. One of the simplest methods defines a non-focal pattern when the LUS score calculated in the anterior lung regions exceeds 2 [31]. Once the phenotype has been identified, imaging techniques do not provide guidance on individualized PEEP selection. Clinical studies have often evaluated low versus high PEEP using dichotomous approaches (e.g., 5 vs. 15 cmH_2_O); however, such “all-or-nothing” strategies may result in suboptimal PEEP levels in both responders and non-responders and may increase the risks associated with misclassification and misalignment. In contrast, advanced assessment of respiratory mechanics may support individualized PEEP titration, thereby optimizing ventilator settings to prevent VILI.

### 2.3. Tidal Pressure–Volume Loops to Optimize Mechanical Ventilation Settings

#### 2.3.1. Targets for Mechanical Ventilation in Patients with ARDS/AHRF

In patients with ARDS and AHRF, mechanical ventilation provides life-saving support, but it may also cause further injury and worsen clinical outcomes when ventilator settings are not optimized and individualized to the patient’s needs [2,34].

When setting the ventilator for these patients, priority should be given to preventing VILI rather than optimizing gas exchange alone [35]. The goal is to ensure adequate gas exchange while minimizing the stress applied to lung tissue. SpO_2_ values of 88–95% and an arterial pH ≥ 7.30 are commonly used targets for oxygenation and CO_2_ removal, respectively, in this setting [35,36]. This strategy also helps limit the hemodynamic impact of positive-pressure mechanical ventilation [37].

High stress within lung tissue results primarily from two mechanisms: alveolar overdistention and the cyclic opening and closing of lung units (small airways ± alveoli), also termed tidal recruitment/derecruitment [35]. Overdistention occurs mainly in the most non-dependent regions of the lung, where distending pressure (i.e., end-inspiratory transpulmonary pressure: alveolar pressure minus pleural pressure) is highest. Clinical measurement of transpulmonary pressure (PL) relies on esophageal pressure monitoring, which requires specific expertise [38,39] and is not available in many clinical settings [40]. In everyday clinical practice, when PL monitoring is unavailable, limiting plateau pressure (Pplat, the end-inspiratory alveolar pressure) to <30 cmH_2_O is a recommended pragmatic approach for reducing excessive lung stress [1,36]. However, because Pplat reflects the mechanics of the entire respiratory system, it does not correspond to a fixed PL value [40]. In patients with abnormal chest wall (e.g., a heavier and/or stiffer chest wall due to morbid obesity, intra-abdominal hypertension, or chest deformity), pleural pressure may be substantially higher and, for the same Pplat, transpulmonary pressure correspondingly lower. Therefore, limiting Pplat to <30 cmH_2_O may expose these patients to unnecessarily low tidal volume and hypoventilation. In such cases, a higher Pplat threshold may be used, but esophageal pressure monitoring to determine the actual PL is strongly recommended [41].

Tidal recruitment/derecruitment of lung units occurs when PL falls below zero in distal small airways during expiration, promoting their collapse, and when the subsequent inspiratory increase in PL is sufficient to reopen them [35,42,43]. Therefore, lung driving pressure (DP)—that is, the difference between end-inspiratory PL (the recruiting pressure) and end-expiratory PL (the pressure opposing collapse)—is the main determinant of tidal recruitment/derecruitment. In clinical practice, limiting airway DP, i.e., the inspiratory increase in alveolar pressure, is considered a useful surrogate for limiting or preventing the cyclic opening and closing of lung units. Notably, airway DP can be reduced by decreasing Pplat and/or increasing PEEP. Tidal recruitment/derecruitment may involve both distal airways and alveoli, especially in the most dependent lung regions [44]. However, in most cases, gas remains trapped distal to the collapsing airways during expiration, and complete reabsorption atelectasis is prevented by the subsequent reopening of these airways [43,44,45]. Thus, cyclic opening and closing occur mainly in poorly—or even normally—aerated lung tissue, consistent with VILI-related inflammation being located predominantly in the central lung regions [46,47].

#### 2.3.2. Tidal Pressure–Volume Loops as a Diagnostic Tool

Lung overdistention is associated with a decrease in lung tissue density that can be detected by quantitative CT. Conventionally, lung tissue with a CT density ranging from −1000 to −900 HU is considered overdistended. Quantitative CT defines tidal recruitment/derecruitment as the amount of non-aerated lung tissue (−100 to +100 HU) at end-expiration that regains some degree of inflation (any density value <−100 HU) at end-inspiration [48]. Thus, CT can detect and quantify tidal recruitment/derecruitment only when it involves lung units located within non-aerated lung tissue. Because most tidal recruitment/derecruitment appears to occur in poorly—or even normally—aerated lung tissue, quantitative CT is not the optimal tool for assessing this mechanism of increased lung stress and VILI. Moreover, quantitative CT is unsuitable for the daily management of mechanically ventilated patients with ARDS/AHRF because it requires transport to the radiology department and involves substantial radiation exposure.

Quasi-static pressure–volume (PV) loops provide information on the sequential opening and closing of lung units: the greater the loop hysteresis (i.e., the area enclosed by the loop), the greater the extent of lung recruitment/derecruitment [42,43,49,50,51,52]. Notably, implementation of this approach is device-dependent because a slow inflation and deflation maneuver is required to obtain a quasi-static pressure–volume loop. When this functionality is available, slow inflation–deflation maneuvers allow airway pressure to approximate alveolar pressure by minimizing the resistive pressure component. The maneuver can be tailored to the range of alveolar pressures occurring during tidal ventilation; these “tidal” PV loops provide information on both tidal overdistention and recruitment/derecruitment [3,12]. For this purpose, the respiratory system is slowly inflated from PEEP to Pplat and subsequently deflated back to PEEP. This highly informative maneuver is simple, rapid (10–15 s), and safe, making it a practical bedside tool for assessing mechanically ventilated patients with ARDS/AHRF.

Tidal hysteresis is measured as the average volume difference between the inflation and deflation limbs. It therefore represents a fraction of tidal volume and can also be normalized to the patient’s predicted body weight (PBW) and expressed in mL/kg. Thresholds for tidal hysteresis have been proposed and evaluated in adult patients with ARDS: values greater than 100 mL or 1.4 mL/kg of PBW are associated with substantial tidal recruitment/derecruitment of lung units [13,14]. The 100 mL threshold was derived post hoc in 38 passively ventilated patients with moderate-to-severe COVID-19 ARDS undergoing a decremental PEEP trial. In these patients, a tidal PV loop was obtained at each PEEP level by slowly inflating and deflating the respiratory system between PEEP and plateau pressure at a constant rate of pressure increase/decrease (2 cmH_2_O/s). Using a stepwise hysteresis increase ≥10 mL/cmH_2_O as the physiological reference, this threshold achieved an area under the curve of 0.97, with 83% sensitivity and 96.5% specificity [13]. In a separate cohort of 50 patients with ARDS, including COVID-19 and non-COVID-19 cases, the 100 mL threshold showed strong agreement with the same criterion (κ = 0.88), while the PBW-normalized threshold achieved 94% sensitivity and 88% specificity [14].

The average slope of the tidal PV loop corresponds to the tidal (chord) compliance of the respiratory system [3]. Measurement of compliance is affected by cyclic opening-closing that may falsely increase its value; tidal hysteresis can also be used to check the reliability of this measurement. Once tidal recruitment/derecruitment has been excluded at baseline, lung overdistention is suggested by a decrease in tidal compliance following a change in ventilator settings that increases Pplat. The inflation limb may provide further insight into the patient’s respiratory mechanics: a progressive decrease in its slope (i.e., UIP: upper inflection point) suggests tidal overdistention, provided that tidal recruitment/derecruitment is absent. In addition, a flat initial portion of the inspiratory limb indicates airway closure or intrinsic PEEP, with no lung inflation occurring until airway pressure reaches either the airway opening pressure (AOP) or a value equal to total PEEP (external PEEP + intrinsic PEEP). Bedside detection of airway closure and measurement of AOP and total PEEP by quasi-static PV loops is clinically relevant because it allows reliable measurement of both driving pressure and compliance of the respiratory system. In fact, when complete airway closure or dynamic hyperinflation is present, the real driving pressure (i.e., the inspiratory increase in alveolar pressure) is better represented by the difference between plateau pressure and AOP or total PEEP (instead of set PEEP). This also applies when esophageal pressure monitoring is performed: inability to detect and quantify AOP may result in substantial overestimation of transpulmonary pressure and underestimation of lung compliance.

#### 2.3.3. Performing and Interpreting PEEP Trials: The Combined Approach

In mechanically ventilated patients with ARDS/AHRF who are hemodynamically stable and show evidence of lung recruitability, a recruitment maneuver followed by PEEP optimization may be considered under close respiratory and hemodynamic monitoring [1]. Published studies indicate that transient hypotension and oxygen desaturation are the most common adverse events during recruitment maneuvers, whereas serious adverse events, including arrhythmias and barotrauma, are uncommon [1]. Within the proposed framework, the maneuver should be discontinued immediately if a predefined safety criterion is met, including hemodynamic instability or escalating vasopressor support, a significant arrhythmia, severe oxygen desaturation, suspected pneumothorax, new or worsening right ventricular dysfunction, and increased intracranial pressure.

Extended PV loops, usually obtained over a pressure range of 0–40 cmH_2_O, may be used to estimate lung recruitability [52]. A tidal PV loop showing cyclic opening and closing of lung units may itself indicate potential for recruitment and prompt reassessment of the ventilator settings. Accordingly, obtaining a tidal PV loop at the initial ventilator settings may reduce the need for more extensive maneuvers, such as extended PV loops, which expose the respiratory system to a broader pressure range and may promote substantial lung collapse and/or overdistention in an already critically ill patient.

To optimize ventilator settings, a decremental PEEP trial can be performed: conventionally, PEEP is first set at a high level and then reduced every 2–3 min in small steps (2 cmH_2_O each). According to the “best compliance approach,” the PEEP level associated with the highest compliance represents the best compromise between lung overdistention and collapse [53,54]. However, in addition to being a mechanism of lung injury, tidal recruitment/derecruitment can also complicate the interpretation of the trial. Indeed, an increase in measured compliance after a PEEP step-down may reflect insufficient PEEP, resulting in tidal recruitment/derecruitment [13]. Therefore, compliance alone can be misleading and may suggest a PEEP level that is lower than optimal. The so-called “combined approach” is a refinement of the previously described “best compliance” method. It is based on the measurement of tidal compliance and hysteresis by generating a tidal PV loop at the end of each PEEP step, as previously described [13,14]. Within this proposed approach, the best PEEP is defined as the value associated with the highest compliance and low tidal hysteresis (i.e., <100 mL or <1.4 mL/kg PBW). The trial can conveniently be stopped as soon as respiratory system compliance decreases and/or tidal hysteresis rises above the threshold.

### 2.4. Detecting Complications Using Ultrasound

Once recruitment maneuvers have been performed and PEEP has been set, a comprehensive ultrasound examination may also facilitate the early detection of potential complications associated with higher intrathoracic pressures.

#### 2.4.1. Pneumothorax and Hyperinflation

Pneumothorax can be readily ruled out by identifying lung sliding, lung pulse, B-lines, or consolidations/pleural effusions [8]. Its pathognomonic ultrasound sign is the lung point [55], which confirms the diagnosis with high specificity and outperforms chest radiography [56]. Its surface extent can be estimated by mapping the lung point on the thorax; in free-air collections, the farther laterally the lung point is located, the greater the percentage of collapsed lung [57]. This semiquantitative assessment should be interpreted with caution in complex, inflamed lungs exposed to prolonged mechanical ventilation, where pleural adhesions may be present and confound its interpretation. In clinically stable patients, CT should be considered as a second-line imaging modality [10].

Absent or reduced lung sliding is a nonspecific finding and may be observed in several conditions, including pneumothorax, apnea, pleural adhesions, mainstem intubation, and lung overdistension. Therefore, additional ultrasound findings have been explored to better identify overdistension, including qualitative identification of a prominent A-pattern, changes in lung sliding quantified by pleural strain, and speckle-tracking analysis [58,59]. Recent findings have shown that assessment of pleural line motion using open-source artificial intelligence software accurately detects regional overdistension when compared with electrical impedance tomography (EIT) [60].

#### 2.4.2. Hemodynamic Impact

Right ventricular (RV) failure occurs in 22–50% of patients with ARDS [61,62]. ARDS increases pulmonary vascular resistance through hypoxic pulmonary vasoconstriction, hypercapnia, microthrombosis, and vasoregulatory imbalance [63]. The resulting increase in pulmonary vascular resistance increases RV afterload, leading to acute RV dilation, tricuspid regurgitation, and impaired left ventricular filling due to septal shift, potentially resulting in hypotension and shock [64]. Echocardiography plays a central role in the bedside assessment of RV function, enabling early identification of acute cor pulmonale. This condition is characterized by RV dilation (RVEDA/LVEDA > 0.6) and septal dyskinesia and reflects acute RV failure secondary to increased afterload [65]. Its presence has been associated with increased mortality in ARDS and COVID-19 [66,67], underscoring the need for prompt recognition and targeted management. Positive-pressure ventilation may precipitate or aggravate this condition by increasing RV afterload; therefore, RV function should be monitored before and after higher PEEP levels are applied. Hypercapnia and hypoxemia further increase pulmonary vascular resistance and are detrimental to RV function. In this setting, prone positioning may mitigate RV stress by improving oxygenation, reducing PaCO_2_, and lowering ventilatory pressures; it has also been shown to reduce mortality in ARDS [68].

ARDS-associated increases in right heart pressures may reverse the physiological interatrial pressure gradient, promoting right-to-left shunting through a patent foramen ovale (PFO) and thereby worsening systemic hypoxemia. Despite the implementation of lung-protective ventilation, PFO has been reported in 11–19% of patients with ARDS [69,70]. A paradoxical worsening of oxygenation after PEEP escalation should prompt consideration of an intracardiac right-to-left shunt [71]. Diagnosis is based on echocardiographic assessment, including color Doppler and contrast-enhanced imaging with agitated saline; provocative maneuvers such as the Valsalva maneuver or transient PEEP elevation may improve the sensitivity of shunt detection [72].

Transesophageal echocardiography has long been considered the reference technique for hemodynamic monitoring in the prone position [73,74]. More recently, particularly during the COVID-19 pandemic, transthoracic echocardiography has emerged as a feasible and useful alternative, with dedicated techniques enabling reliable hemodynamic assessment, particularly of the RV, even in patients in the prone position [75,76,77].

### 2.5. Monitoring and Optimization of Mechanically Ventilated Patients

#### 2.5.1. Fine-Tuning and Monitoring of Mechanical Ventilation Using Tidal PV Loops

**Figure 2:**
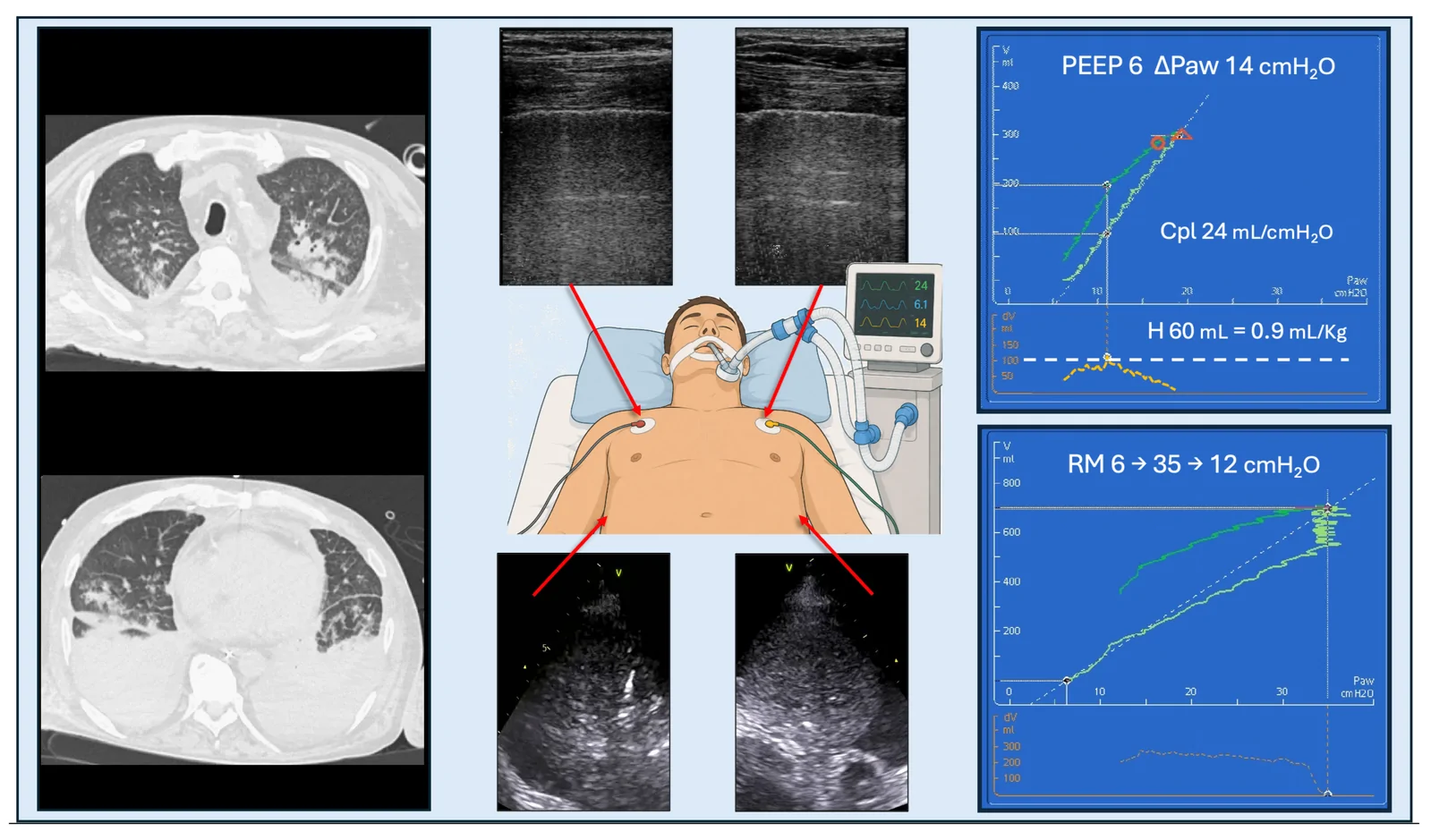
Example of patient assessment in the supine position. (**Left**) the CT scan shows a focal distribution of aeration loss. (**Middle**) the corresponding lung ultrasound findings demonstrate normal aeration (score 0) in the anterior fields and lobar consolidations (score 3) in the posterior fields. (**Top right**) given the low recruitability suggested by the focal lung morphology, a low PEEP (6 cmH_2_O) was set. The tidal PV loop shows low compliance (Cpl = 24 mL/cmH_2_O) but normal hysteresis (60 mL; 0.9 mL/kg), thus ruling out cyclic opening and closing of lung units despite the low PEEP and ΔPaw_tidal of 14 cmH_2_O. (**Bottom right**) a recruitment maneuver (RM) is performed by increasing airway pressure to 35 cmH_2_O. The resulting volume increase (700 mL) closely matches the value predicted from compliance (predicted volume increase = Cpl × ΔPaw_RM = 24 mL/cmH_2_O × 29 cmH_2_O = 696 mL). This finding is consistent with low recruitability in the supine position, as predicted by lung morphology. The patient remains severely hypoxemic, with a PaO_2_/FiO_2_ ratio of 100 mmHg despite an FiO_2_ of 1.0; therefore, prone positioning is initiated (see Figure 3). The PV loops shown in this figure were obtained using a Hamilton G5 ventilator (Hamilton Medical AG, Bonaduz, Switzerland); lung ultrasound images were obtained with a Vivid-IQ GE Healthcare (Chicago, IL, USA).

**Figure 3:**
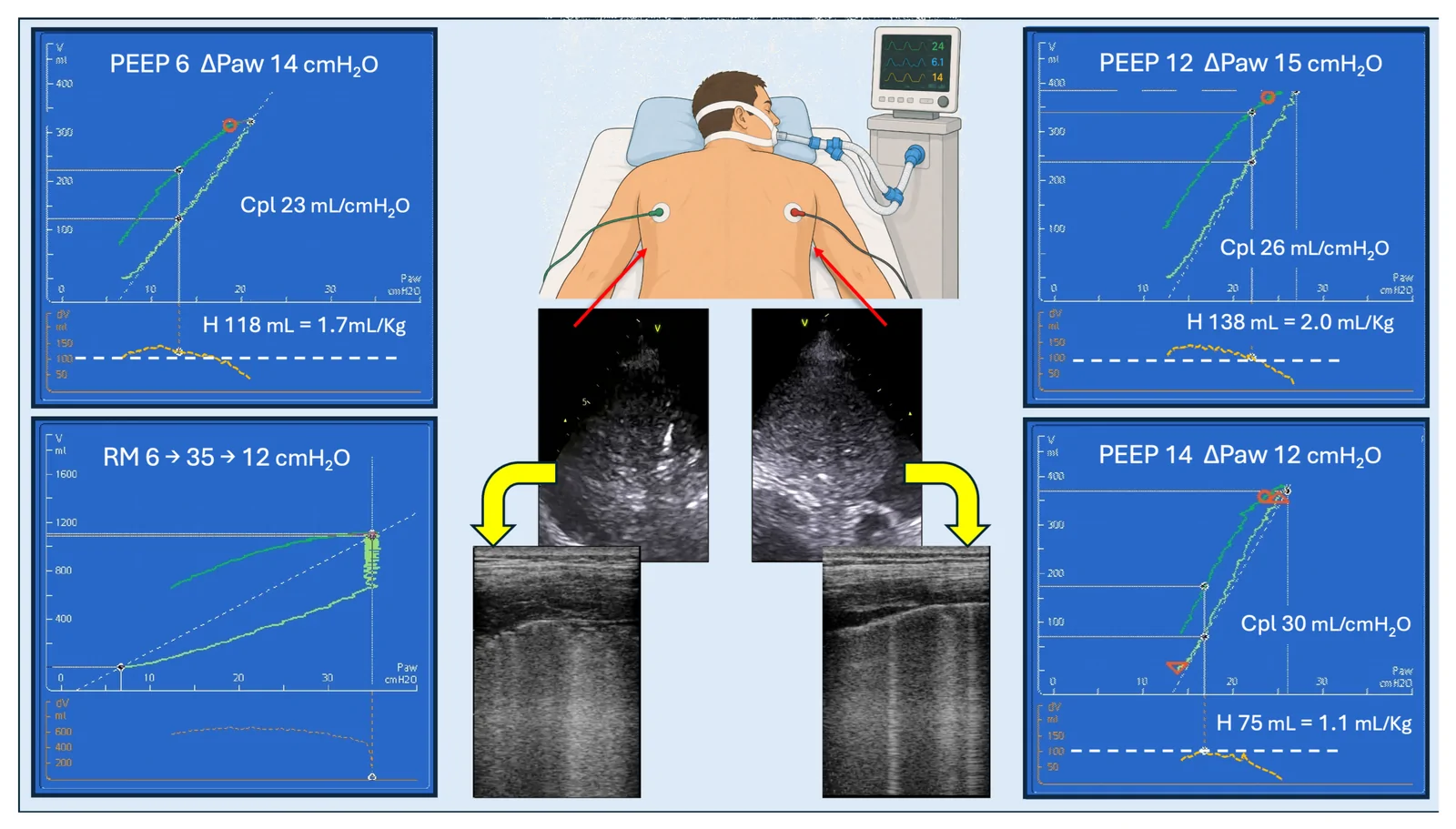
After prone positioning, the same patient could present a different pattern. Reaeration of the posterior fields is detected, with a transition from a tissue-like pattern (score 3) to moderate loss of aeration (score 2) on the left and mild loss of aeration (score 1) on the right. (**Top left**) the first tidal PV loop, performed at a PEEP of 12 cmH_2_O and a driving pressure of 14 cmH_2_O, shows similar compliance (Cpl = 26 mL/cmH_2_O) but greater tidal hysteresis (138 mL; 2.0 mL/kg PBW), suggesting cyclic opening-closing and potential for recruitment. (**Bottom left**) this corresponds to a greater volume reached (1100 mL) during an otherwise identical recruitment maneuver (RM), in which airway pressure is increased from 6 to 35 cmH_2_O and then reduced to 12 cmH_2_O. (**Right**) after fine-tuning, PEEP was set at 14 cmH_2_O with a driving pressure of 12 cmH_2_O, resulting in hysteresis < 100 mL. The PV loops shown in this figure were obtained using a Hamilton G5 ventilator (Hamilton Medical AG, Bonaduz, Switzerland); lung ultrasound images were obtained with a Vivid-IQ GE Healthcare (Chicago, IL, USA).

Tidal PV loops can be generated regularly in mechanically ventilated patients to help clinicians optimize ventilator settings. A simple closed-loop algorithm has been proposed [3]. Briefly, a tidal PV loop is obtained to identify the presence or absence of cyclic opening and closing of lung units and overdistention. Based on these findings, small adjustments to PEEP and/or driving pressure (or tidal volume) are made. A new tidal PV loop is then generated to assess the effects of the new settings. This process is repeated until protective ventilator settings are achieved. The protocol is intended to reduce the risk of VILI and limit the amount of energy dissipated into the lung tissue during tidal ventilation. Notably, tidal PV loops can be used to apply both an “open lung” or a “permissive atelectasis” strategy, thus providing physicians with the opportunity to personalize their strategy in ARDS patients. This simple, safe, and rapid algorithm can be performed daily as a routine procedure to assess and optimize ventilator settings, as well as whenever the patient’s condition changes. Figure 2 and Figure 3 provide illustrative clinical examples of the combined approach based on ultrasound and PV loops and are intended to demonstrate its physiological rationale and practical implementation. A tidal PV loop can also provide a “snapshot” of the mechanically ventilated patient, encompassing several parameters: ventilator settings (PEEP, driving pressure, Pplat, and Vt/kg), respiratory mechanics (tidal compliance of the respiratory system and hysteresis), and ventilatory energetics (total and dissipated mechanical energy). These snapshots can be conveniently stored in the ventilator and/or incorporated into digital medical reports to facilitate trend analysis and monitoring [3].

#### 2.5.2. Lung Aeration and Recovery

Unlike CT, which is impractical for repeated assessments, LUS enables serial, real-time evaluation of lung aeration without requiring the transport of critically ill patients [78,79].

Once PEEP has been optimized, changes in the LUS aeration score can be used to monitor its effects on global and regional lung aeration. Variations in the score have been shown to correlate with PEEP-induced lung recruitment as assessed by PV loops, EIT, and the recruitment-to-inflation ratio, as well as with improvements in gas exchange [80,81,82,83]. This lung recruitment mainly reflects air recruitment, defined by changes in tissue properties at different lung volumes. In contrast, no significant correlation has been found between changes in the LUS aeration score and PEEP-induced tissue recruitment measured by CT, which is defined as the reopening of previously collapsed alveoli. Because the LUS aeration score detects any improvement in aeration—including changes in regions previously assigned a score of 1 or 2—these two measurements assess different physiological phenomena and may therefore not correlate.

LUS also enables bedside monitoring of regional changes in aeration during prone positioning, with the most relevant changes occurring in the posterior lung regions. Re-aeration of these regions, as assessed by LUS, correlates with improved gas exchange and reduced dead space [83,84]. Significant re-aeration of the posterior lung fields during the first prone positioning cycle has been associated with better outcomes and sustained improvement in the PaO_2_/FiO_2_ ratio [84]. Additionally, the magnitude of the reduction in dead space has been shown to correlate with improvements in LUS aeration score during prone positioning [84]. Posterior aeration gain detected by LUS correlates with increases in end-expiratory lung volume (EELV), as measured by nitrogen washout/washin methods [79]. Variations in the LUS aeration score throughout the prone positioning session have been significantly associated with the response to prone positioning in terms of both lung inflation and oxygenation [79]. However, neither LUS nor changes in EELV detect hyperinflation; therefore, LUS should not be used in isolation to guide respiratory management [79].

Daily lung imaging is essential in the management of ARDS, both for evaluating disease progression and for the early identification of complications. LUS provides a bedside alternative to traditional imaging, enabling frequent quantitative monitoring of lung aeration using the LUS aeration score. This is particularly relevant in patients with severe ARDS, especially those on extracorporeal membrane oxygenation (ECMO), for whom transport for CT carries significant risks [85]. Whereas lung recovery corresponds to a progressive reduction in the LUS aeration score [85,86,87], an unexpected increase reflects decreased aeration [88] and may indicate disease progression or the onset of complications such as ventilator-associated pneumonia (VAP) [89]. Early identification of complications such as VAP is critical in mechanically ventilated patients, and LUS plays a key role in this setting. Two ultrasound signs have been associated with VAP: subpleural consolidations and a dynamic linear-arborescent air bronchogram within a consolidation [89,90]. In ARDS patients, subpleural consolidations are a common finding; therefore, their presence alone is insufficient to diagnose VAP. In contrast, a newly appeared dynamic linear-arborescent air bronchogram provides high specificity and enables earlier diagnosis when VAP is clinically suspected [89,90,91]. The specificity of this sign when identified in a single lung region has been reported to be 81% [90].

A summary of ultrasound and pressure-volume findings and their corresponding clinical action is reported in Table 1.

## 3. Limitations and Practical Considerations

Several limitations should be considered when applying the proposed approach. Reliable acquisition of tidal PV loops requires passive respiratory conditions throughout the slow inflation–deflation maneuver. Consequently, the PV-loop component cannot be applied to patients breathing spontaneously during assisted ventilation or weaning, although LUS and hemodynamic ultrasound remain applicable in these settings. As discussed above, implementation is also limited to ventilators capable of performing a slow inflation–deflation maneuver and acquiring both limbs of the resulting PV loop. Moreover, we are not aware of studies comparing different tools to generate quasi-static PV loops: whether the same maneuver may generate different values when performed with different mechanical ventilators cannot be excluded. Finally, significant gas leaks, such as those caused by a bronchopleural fistula or an active leak through a chest-drainage system, interfere with volume measurement and preclude reliable quantification of hysteresis.

**Figure 4:**
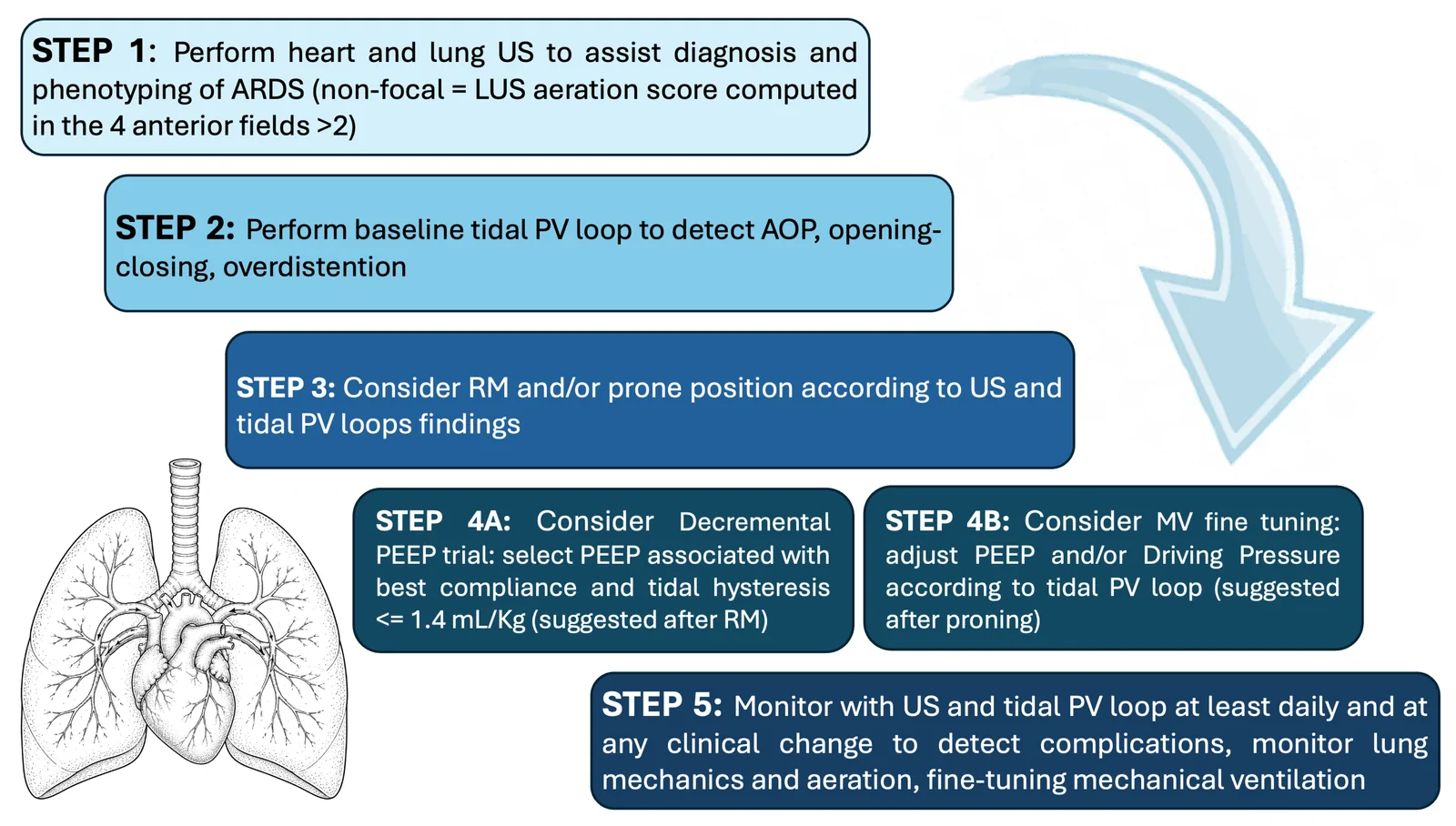
A physiology-based framework combining lung ultrasound findings, tidal pressure–volume loops, and hemodynamic assessment. (US: ultrasound, LUS: lung ultrasound; ARDS: Acute respiratory distress syndrome; tidal PV loop: pressure–volume loop from PEEP to Plateau pressure; AOP: airway opening pressure, RM: recruitment maneuver; MV: mechanical ventilation).

Like other ultrasound techniques, LUS assessment remains operator dependent. Nevertheless, studies have demonstrated excellent interobserver agreement in the assessment of basic findings and lung aeration when examinations are performed by experienced operators [92,93]. Adequate training and hands-on experience are therefore essential to ensure accurate image acquisition and interpretation and the reliable integration of LUS findings into clinical management. In patients with morbid obesity or markedly altered chest wall mechanics, image acquisition may be more challenging, and airway pressures may not accurately reflect the pressure effectively distending the lung. Esophageal manometry can help partition respiratory-system mechanics into their lung and chest wall components. However, esophageal pressure remains an indirect estimate of pleural pressure and is influenced by balloon position, filling volume, calibration, body position, and local mediastinal effects [38,39,40]. Furthermore, it provides only a regional surrogate of pleural pressure and cannot fully capture its spatial heterogeneity. Finally, the proposed algorithm (Figure 4) should be regarded as a physiology-based framework that complements comprehensive clinical assessment; its effect on patient-centered outcomes requires prospective validation.

## 4. Conclusions

An integrated approach combining lung ultrasound imaging, assessment of respiratory mechanics using pressure–volume loops, and hemodynamic evaluation enables a comprehensive, non-invasive bedside assessment of patients with acute respiratory failure, potentially improving the characterization of lung physiology and facilitating personalized ventilatory strategies.

## Figures and Tables

**Table 1:** Findings, interpretation and corresponding clinical action for ultrasound and pressure–volume loops.

Findings	Interpretation	Corresponding Clinical Action
Lung ultrasound		
LUS aeration score computed in anterior fields ≤ 2	Focal distribution of loss of aeration—limited potential for recruitment	Consider prone positioning to improve patient’s oxygenation
LUS aeration score computed in anterior fields > 2	Non-focal distribution of loss of aeration—potential for recruitment	Consider performing a decremental PEEP trial to improve patient’s oxygenation
Lung point	Pneumothorax	Stable patient: monitor the localization of the lung point to promptly identify its expansion; eventually consider CT scan Unstable patient: consider pleural drainage
Reduced/absent lung sliding	Compatible with overdistention Absent sliding may be a pneumothorax; move laterally and inferiorly searching for a lung point	Consider reducing intrathoracic pressure
Transthoracic echocardiography		
RVEDA/LVEDA > 0.6 and/or septal dyskinesia	Acute RV failure	Consider reducing intrathoracic pressure or modifying factors increasing RV afterload (acidosis, oxygenation, vasoconstriction)
Positive bubble test after PEEP increase	PEEP-induced right-to-left shunt	Consider reducing intrathoracic pressure or modifying factors increasing RV afterload (acidosis, oxygenation, vasoconstriction)
PV loops from PEEP to Plateau Pressure		
Initial horizontal part of the inspiratory limb (increase in pressure without increase in volume)	Complete airway closure with AOP higher than PEEP	Consider AOP to calculate driving pressure and compliance; consider increasing PEEP
Large PV loop: Tidal Hysteresis > 1.4 mL/kg	Expiratory collapse with cyclic opening-closing of lung units (small airways ± alveoli)	Consider increasing PEEP (open lung) and/or reducing driving pressure (permissive atelectasis) to minimize VILI
Small PV loop (tidal Hysteresis ≤ 1.4 mL/kg) with protective DP and Pplat values	Protective ventilation	Confirm MV setting or consider start of PEEP weaning
Small PV loop + UIP or decrease in Crs when increasing PEEP/DP starting from a small PV loop	Overdistention	Consider reducing PEEP and/or driving pressure

LUS: lung ultrasound; CT: computed tomography; RVEDA: right ventricular end-diastolic area; LVEDA: left ventricular end-diastolic area; RV: right ventricle; PEEP: positive end-expiratory pressure; AOP: airway occlusion pressure; VILI: ventilator-induced lung injury; UIP: upper inflection point; Pplat: plateau pressure; MV: mechanical ventilation; PV: pressure–volume; Crs: compliance of the respiratory system; DP: driving pressure.

## Data Availability

No new data were created or analyzed in this study. Data sharing is not applicable to this article.

## Abbreviations

The following abbreviations are used in this manuscript:

AHRF	Acute hypoxemic respiratory failure
AOP	Airway opening pressure
ARDS	Acute respiratory distress syndrome
Crs	Compliance of the respiratory system
CT	Computed tomography
DP	Driving pressure
E/e′	Early transmitral flow velocity early diastolic mitral annular velocity ratio
ECMO	Extracorporeal membrane oxygenation
EELV	End-expiratory lung volume
EIT	Electrical impedance tomography
ICU	Intensive care unit
LUS	Lung ultrasound
LV	Left ventricular
LVEDA	Left ventricular end-diastolic area
PaCO_2_	Partial pressure of arterial carbon dioxide
PaO_2_	Partial pressure of arterial oxygen
PEEP	Positive end-expiratory pressure
PFO	Patent foramen ovale
PL	Transpulmonary pressure
Pplat	Plateau pressure
PV loop	Pressure–volume loop
RV	Right ventricular
RVEDA	Right ventricular end-diastolic area
UIP	Upper inflection point
VAP	Ventilator-associated pneumonia
VILI	Ventilator-induced lung injury
